# A Unified Multispectral Feature Framework for Independent Recognition of Floods, Burned Areas, and Landslides

**DOI:** 10.3390/s26216942

**Published:** 2026-11-03

**Authors:** Leqiang Zhong, Wenzhi Han, Yuhui Feng, Qiaochu Yang, Zhi Liu, Peng Zhang

**Affiliations:** School of Optoelectronic Engineering, Changchun University of Science and Technology, Changchun 130022, China; 2024003295@mails.cust.edu.cn (L.Z.); 2024000911@mails.cust.edu.cn (W.H.); 2024003023@mails.cust.edu.cn (Y.F.); yangqc@cust.edu.cn (Q.Y.); liuzhi@cust.edu.cn (Z.L.)

**Keywords:** multispectral remote sensing, shared feature representation, flood, burned area, landslide, grouped evaluation, independent experts

## Abstract

Multispectral disaster recognition draws on common optical measurements, but differences in feature construction, target definitions and evaluation design complicate comparisons across tasks. We present a unified feature framework that applies the same six-band interface, 78-dimensional spectral–spatial descriptor and training-side learning procedure to independently fitted flood, burned area and landslide experts. Spectral summaries, local variability and gradients describe each window; paired date and source auxiliary inputs provide task-specific extensions. Evaluation covered 88 flood patches from 11 events, 656 burned area windows in 23 source/overlap groups, and a prespecified 100-patch subset of the official Landslide4Sense test partition. The shared representation achieved F1 values of 0.8504, 0.8485 and 0.8169, respectively, under training-side classifier selection. The burned area temporal extension increased F1 to 0.8997, with gains over matched support post-event descriptors under both inner-selected and fixed Random Forest evaluation. The landslide auxiliary extension produced a larger gain under classifier selection than under fixed Random Forest, identifying an interaction between representation and classifier. Training label controls and negative class metrics distinguished learned discrimination from positive class prevalence. The framework provides a reproducible basis for testing shared optical descriptors and task-specific information under the distinct evaluation requirements of each task.

## 1. Introduction

Floods, wildfires and landslides create a sustained need for spatial information about disturbed land surfaces. The CRED/UNDRR assessment of 2000–2019 recorded 7348 natural hazard disasters, approximately 1.23 million deaths and US$2.97 trillion in reported economic losses, expressed in 2019 dollars. Floods represented 44% of recorded events, while landslides and wildfires accounted for approximately 5% and 3%. These statistics describe threshold-qualified events recorded by EM-DAT across natural hazards collectively [1]. Multispectral observations offer a common measurement basis for examining the surface changes associated with these hazards. Sentinel-2’s visible, near-infrared (NIR) and shortwave infrared (SWIR) bands sample water, vegetation, moisture and exposed ground responses at complementary wavelengths [2].

The spectral mechanisms differ among the three tasks. Water commonly has low NIR and SWIR reflectance, motivating green–SWIR contrasts such as the modified normalized difference water index (MNDWI) [3]. Burned area analysis combines post-fire surface response with changes in vegetation and moisture, traditionally summarized using NIR–SWIR contrasts and pre/post comparisons [4,5]. Landslide inventories combine vegetation disturbance and exposed materials with spatial context; the Landslide4Sense benchmark provides optical and auxiliary layers for pixel-level landslide mapping [6]. These differences motivate task-specific information, while their shared optical channels make it possible to test a common description of spectral distributions and local structure.

Existing machine learning and deep learning studies establish strong task-specific approaches. Sen1Floods11 supplies georeferenced imagery and water annotations for evaluating flood algorithms [7]. The published FLOGA benchmark compares spectral index rules, conventional classifiers and deep change detection models using bi-temporal fire imagery [8]. BiAU-Net learns burned area masks from paired Sentinel-2 imagery and evaluates geographically separate areas [9]. Landslide4Sense benchmarks multiple deep segmentation architectures using optical and auxiliary layers [6]. However, the pixel-wise index and conventional classifier baselines in FLOGA do not explicitly incorporate neighborhood information [8].

Representation sharing also takes different forms. Prithvi reuses or fine-tunes a pretrained multispectral encoder and learns downstream decoders for tasks including flood and wildfire scar segmentation [10]. Its downstream performance reflects representation learning and task-specific adaptation. Across hazard-specific studies, differences in inputs, representations and evaluation designs complicate attribution of performance gains to common optical information [6,8,9,10]. A fixed descriptor protocol offers a complementary form of sharing: measurement channels and aggregation operators remain common, while each task has its own fitted classifier. This makes the contribution of additional observations testable without requiring shared model parameters. Group-aware evaluation is essential because nearby or source-related samples can otherwise give an optimistic impression of transfer to new observations [11].

Two research questions motivate the present study:

- Can a unified spectral–spatial representation provide an effective common feature basis for the independent recognition of floods, burned areas, and landslides?
- How much do local spatial summaries and additional temporal or auxiliary inputs contribute under the same training-side model selection procedure and a fixed classifier comparison?

We address these questions with a unified multispectral feature framework for independent image window presence recognition. Unification operates at the six-band input interface, descriptor implementation, support rules and training-side evaluation procedure. The models remain independently fitted for each hazard. The study contributes a reproducible common descriptor, controlled comparisons of shared and task-specific representations, and an evaluation that keeps source grouping, class composition and cross-source response visible. The resulting evidence concerns three independent recognition tasks, with each task retaining its own reference annotations and evaluation population.

## 2. Materials and Methods

### 2.1. Framework and Prediction Target

**Figure 1:**
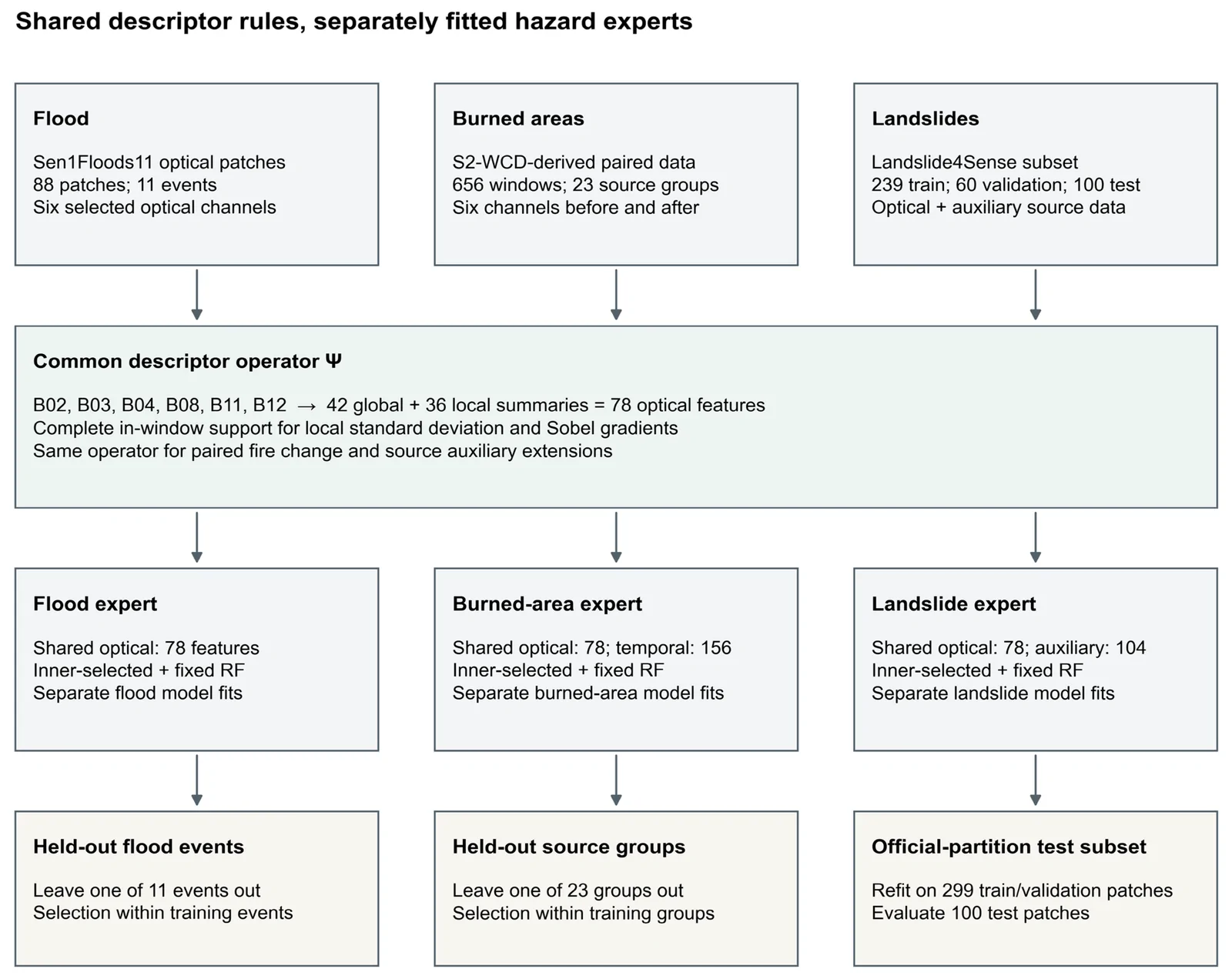
Unified descriptor construction and independent hazard experts. Source-specific arrays enter the same optical descriptor operator. The burned area branch additionally describes paired date change, while the landslide branch can include two source auxiliary channels. Each hazard independently uses the same training-side selection procedure and a fixed Random Forest comparator. Held-out evaluation follows the source-specific grouping or official partition roles. The shared elements are the descriptor and evaluation procedures; each expert has separately fitted parameters.

The framework maps a multispectral image window to a binary presence prediction for one target hazard (Figure 1). Each source supplies six optical channels and an image support mask. A common descriptor operator produces global distribution summaries, local variability summaries and gradient summaries. The same training-side preprocessing, weighting and candidate selection rules are then applied independently within each hazard. A fixed Random Forest provides a second, prespecified evaluation role that separates representation changes from changes in classifier selection.

For window i, the target is y_i_ = 1 when at least one target-positive pixel occurs within valid annotation support, and y_i_ = 0 otherwise. Annotation support defines where the reference label is interpretable; image support defines which optical values contribute to descriptors. Reference labels do not select descriptor pixels. This common presence rule aligns the logical target across sources while preserving differences in pixel spacing, window size, positive area fraction and class prevalence. Each output therefore concerns an image unit, rather than a pixel segmentation or mutually exclusive three-hazard category.

### 2.2. Datasets, Labels and Source Separation

The datasets were selected because they provide annotated multispectral inputs and complementary conditions for evaluating shared descriptors. Sen1Floods11 HandLabeled supplies optical imagery, manual water annotations and a permanent water layer with identifiable flood events [7]. S2-WCD supplies paired observations and burned area change annotations [12]. Landslide4Sense supplies multispectral patches, binary masks, auxiliary channels and official partition roles [6]. Their temporal and auxiliary inputs allow for controlled extensions around the same optical basis (Table 1).

The flood cohort contains 88 image patches of 512 × 512 pixels, comprising 64 presence-positive and 24 presence-negative units. Positive reference pixels satisfy LabelHand = 1 and JRCWaterHand = 0. LabelHand = −1 and annotated water excluded by the permanent water rule do not contribute positive flood labels. Image and label grids were checked separately. All patches from the same event were held out together across 11 leave-one-event-out evaluations.

The burned area cohort contains 656 non-overlapping 128 × 128 windows from 41 paired scenes on the stored 20 m grid, with 446 positive and 210 negative windows. All windows with annotated burned pixels enter the presence-positive class, including four windows below the previous 1 ha inclusion threshold. Scenes with documented source relationships or observed spatial overlap were kept together in 23 conservative groups.

The Landslide4Sense cohort contains 399 patches of 128 × 128 pixels, including 291 positive and 108 negative patches. The experiment retains 239 training patches, 60 validation patches and a prespecified 100-patch subset of the official test partition. The test subset comprises 75 positive and 25 negative patches. Candidate selection uses the training and validation members; the final estimator and preprocessing are refitted on their 299-member union before test evaluation.

**Figure 2:**
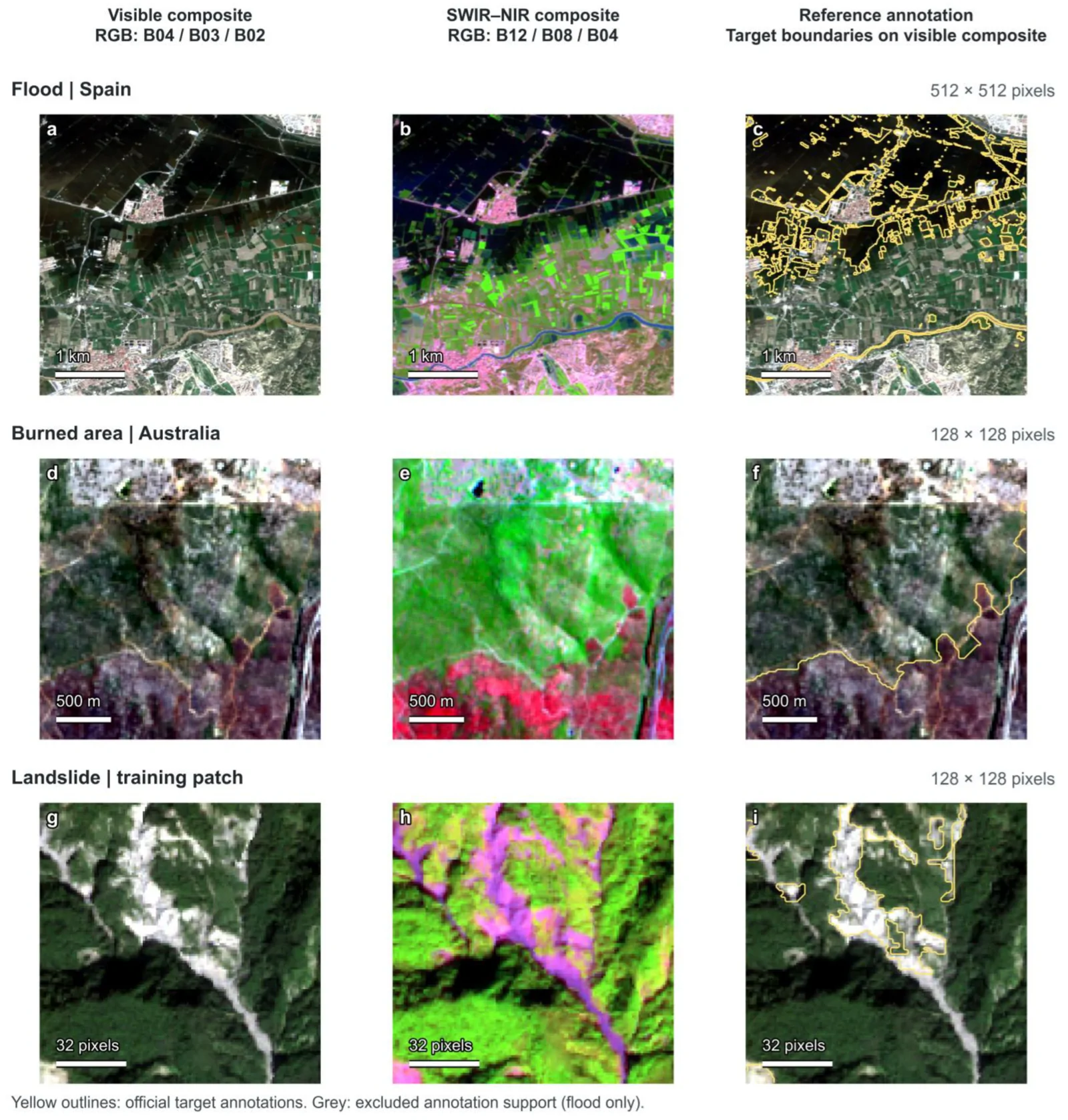
Multispectral image examples and official reference annotations. Rows show flood, burned area and landslide inputs, respectively. (**a**,**d**,**g**) Visible composites use B04/B03/B02 as RGB; (**b**,**e**,**h**) false-color composites use B12/B08/B04; (**c**,**f**,**i**) yellow outlines show the target annotations on the corresponding visible composites. Grey areas denote excluded flood annotation support after permanent water exclusion. The burned area panels show the post-event input. Each row retains the full analysis window and the same extent in all three columns. Each display channel uses its within-window 2nd–98th percentile stretch and gamma 1.15, solely for visualization. Ground distance bars use the supplied grids; the Landslide4Sense bar is in pixels because its H5 array has no georeferencing.

Figure 2 illustrates visible and SWIR–NIR composites of one existing sample from each source, together with the official target annotations.

### 2.3. Optical Inputs and Complete Support Descriptors

The shared optical channel order is B02, B03, B04, B08, B11 and B12, representing blue, green, red, NIR, SWIR1 and SWIR2. Flood digital numbers are divided by 10,000, and image support requires valid raster read masks and finite values in all six channels. Stored burned area and Landslide4Sense arrays enter numerically unchanged, with finite channel support. The numerical scale of each dataset is retained; a common physical reflectance scale across the three processed sources has not been established.

Let Ψ(X, M) denote the descriptor of channel array X on image support M. Seven global statistics are calculated for each band: mean, population standard deviation, and the 10th, 25th, 50th, 75th and 90th percentiles. The six bands therefore yield 42 global descriptors. For each band, local population standard deviation is calculated in 3 × 3 and 7 × 7 neighborhoods. The mean and 90th percentile of each local standard deviation map contribute four descriptors per band. The mean and 90th percentile of Sobel magnitude contribute two further descriptors per band. These 36 local descriptors produce a 78-dimensional optical representation in total.

**Figure 3:**
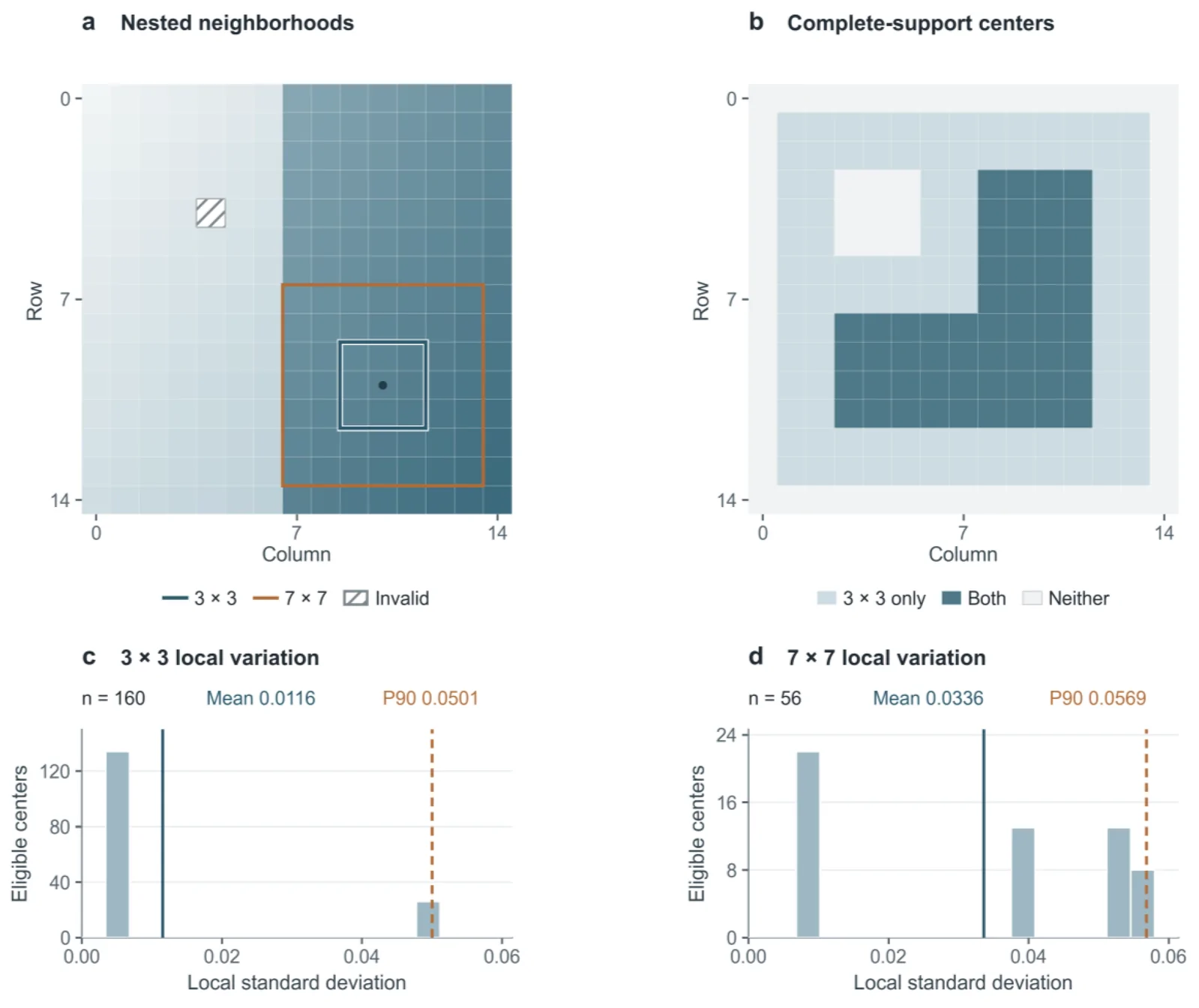
Complete-neighborhood support and local variability aggregation, illustrated using a synthetic 15 × 15 array. (**a**) Nested 3 × 3 and 7 × 7 windows. (**b**) Eligible centers for complete 3 × 3 support only (light blue), both neighborhood sizes (dark blue), or neither (gray). (**c**,**d**) Local standard deviation distributions for the 3 × 3 and 7 × 7 neighborhoods, with 160 and 56 eligible centers, respectively. Solid and dashed lines indicate the mean and 90th percentile. The illustration explains the descriptor operation and does not represent satellite measurements.

Local calculations require a complete in-window support stencil. Array boundaries and invalid pixels are outside support, and values inserted at invalid positions cannot affect a retained neighborhood. Global and local summaries use their corresponding eligible support, and an empty support yields a missing feature for training-side imputation. Figure 3 illustrates how eligible centers produce local standard deviation distributions and their mean and P90 summaries. Neighborhood dimensions are defined in pixels and consequently cover different ground distances on different source grids. The two scales describe fine and broader local variation, consistent with earlier use of local variance across window sizes including 3 and 7 pixels [13].

### 2.4. Controlled Representations and Task-Specific Extensions

Representation comparisons test distinct sources of information (Table 2). The 42-dimensional global-only representation isolates the contribution of local summaries. A 102-dimensional reference combines those global statistics with ten circular local binary pattern histogram bins per band, using eight neighbors at radius one [14]. This is a descriptor adaptation, rather than a reproduction of the original texture classification system. The flood comparisons additionally include seven MNDWI statistics and a 143-dimensional optical-plus-index representation. The latter adds global and local descriptions of NDVI, MNDWI, NBR, NDMI and BSI. These contrasts summarize vegetation, water, moisture and bare ground-related responses; their explicit definitions and valid denominator rules are given in Supplementary Materials. NDVI, MNDWI and NIR–SWIR index use follow the relevant spectral literature [3,4,15,16]. The BSI formula uses the SWIR1-based bare soil contrast summarized by Nguyen et al. [17]. Fixed index fraction thresholds are absent from the common 78-dimensional representation.

The shared spectral indices were defined as follows:

(1) $$\mathrm{NDVI}=\frac{\mathrm{NIR}-\mathrm{Red}}{\mathrm{NIR}+\mathrm{Red}}$$

(2) $$\mathrm{MNDWI}=\frac{\mathrm{Green}-\mathrm{SWIR}1}{\mathrm{Green}+\mathrm{SWIR}1}$$

(3) $$\mathrm{NBR}=\frac{\mathrm{NIR}-\mathrm{SWIR}2}{\mathrm{NIR}+\mathrm{SWIR}2}$$

(4) $$\mathrm{NDMI}=\frac{\mathrm{NIR}-\mathrm{SWIR}1}{\mathrm{NIR}+\mathrm{SWIR}1}$$

(5) $$\mathrm{BSI}=\frac{\left(\mathrm{SWIR}1+\mathrm{Red}\right)-\left(\mathrm{NIR}+\mathrm{Blue}\right)}{\left(\mathrm{SWIR}1+\mathrm{Red}\right)+\left(\mathrm{NIR}+\mathrm{Blue}\right)}$$

For burned areas, the temporal representation is z_temporal_ = [Ψ(X_post_, M_pair_), Ψ(X_post_ − X_pre_, M_pair_)], where M_pair_ is the intersection of valid pre- and post-image support. Both 78-value blocks therefore use the same support. The second block describes the pixelwise post-minus-pre array, including its distributions and local structure. It is different from subtracting two descriptor vectors and is not interpreted as a physically verified dNBR product. A matched support post-only representation and an 84-dimensional concatenation of pre/post global descriptors provide two temporal references.

The landslide extension appends 26 global and local summaries from the two source auxiliary channels to the shared optical vector, yielding 104 descriptors.

### 2.5. Training-Side Selection and Fixed Classifier Evaluation

The candidate grid contrasts randomized tree ensembles with regularized linear classifiers on the same descriptors. Random Forest and ExtraTrees capture nonlinear feature interactions through different randomization mechanisms [18,19], while linear SVM [20] and logistic regression provide regularized linear alternatives. It evaluates the representation under complementary classifiers rather than assigning one algorithm to each hazard in advance. Each hazard uses the same 14 candidates, comprising four Random Forest, four ExtraTrees, three LinearSVC and three logistic regression specifications. Table S1 in the Supplementary Materials lists the parameter configurations.

All preprocessing is fitted within the relevant training partition. Median imputation retains entirely missing columns. Linear models additionally use training-weighted standardization; tree ensembles use imputed, unscaled values. When training groups are available, the initial sample weight is the reciprocal of its group size. Weights are normalized within each class and then divided by their overall mean. When groups are absent, initial weights are one. The same final weights enter estimator fitting and linear model standardization, with estimator class_weight set to None.

Flood and burned area candidate selection uses three inner grouped folds within each outer training set. Candidates are ranked first by balanced accuracy from pooled inner held-out decisions with inverse group size weights, then by the mean group-weighted AP across the same candidate’s inner folds, and finally by model identifier to resolve exact ties. Landslide selection uses the designated validation subset before the final 299-member refit. The term inner-selected denotes this training-side procedure; different outer partitions can select different algorithms or settings. The fixed classifier role always uses a 300-tree Random Forest with minimum leaf size 3 and square root feature sampling, regardless of held-out results.

No probability calibration is applied. Primary decision boundaries are fixed at 0 for the LinearSVC margin and 0.5 for the positive-class outputs of Random Forest, ExtraTrees and logistic regression. Equality is assigned to the positive class. These boundaries keep decision rules fixed during representation comparisons, without selecting thresholds from outer test performance. Scores are not interpreted as calibrated event probabilities [21,22]. Secondary operating points are selected using training-side predictions only. The computational analyses were performed in Python 3.9.1.

### 2.6. Performance, Controls and Sensitivity Analysis

F1, balanced accuracy (BA), precision, recall, specificity, false positive rate (FPR) and confusion counts describe held-out presence decisions. For flood and burned area evaluation, confusion-derived summaries pool held-out class decisions. AUC and average precision (AP) are calculated within held-out groups containing both classes and then macro-averaged, avoiding the pooling of incomparable margins from separately fitted models. Seven of 11 flood groups and all 23 burned area groups support these ranking metrics. Landslide AUC and AP come from the final model on the 100-patch test subset. Undefined single-class group metrics remain unavailable.

Training label controls use 20 permutations per evaluation unit. The classifier configuration selected using the true training labels remains fixed, while the estimator, preprocessing and class-normalized weights are refitted after permuting training labels. Held-out membership and labels remain unchanged. The P2.5–P97.5 interval summarizes the empirical permutation distribution; it is neither a confidence interval nor a permutation test of the entire model selection procedure. Constant-positive predictions provide a prevalence-dependent F1 reference.

Paired representation differences use 2000 resamples of the same fixed held-out predictions [23]. Flood and burned area resampling retains whole event or source groups. Landslide resampling uses test patches and depends on an unverified patch independence assumption. These intervals are conditional on the fitted predictions and exclude refitting and model selection uncertainty. The fixed Random Forest sensitivity analyses vary neighborhood scales, support rules and positive-area requirements. Target retention rates and matched cohort references accompany area threshold results so that changing the evaluated population remains visible.

### 2.7. Cross-Source Responses

Six directions evaluate optical inputs from each source through compatible COMMON78 experts trained for the other two hazards. Only common optical descriptors are transferred. Before transfer, the documented fixed Landslide4Sense optical encoding is converted into the receiving model’s training coordinates (Supplementary Materials, Part C). Every retained receiving model reference evaluates the stated source cohort at its native decision boundary.

Positive response rates summarize transferred cohorts without receiving-hazard annotations. Unique source samples and receiving model references have distinct denominators. Direct stored value transfer provides a numerical encoding sensitivity comparison; training range diagnostics apply to that stored value condition.

## 3. Results

### 3.1. Shared Optical Representation and Negative-Class Recognition

The common descriptor supported all three presence tasks under their designated evaluation designs (Table 3). With training-side selection, shared optical F1 was 0.8504 for flood, 0.8485 for burned area and 0.8169 for landslide. Corresponding BA values were 0.7344, 0.7938 and 0.7067. The fixed Random Forest yielded F1 values of 0.9091, 0.8457 and 0.8493. Reporting both roles preserves the distinction between evaluating the selection procedure and comparing representations at a constant classifier setting.

Class-specific error counts in Table 3 show variation across the held-out populations.

### 3.2. Spectral–Spatial Descriptors and Controlled Comparisons

**Figure 4:**
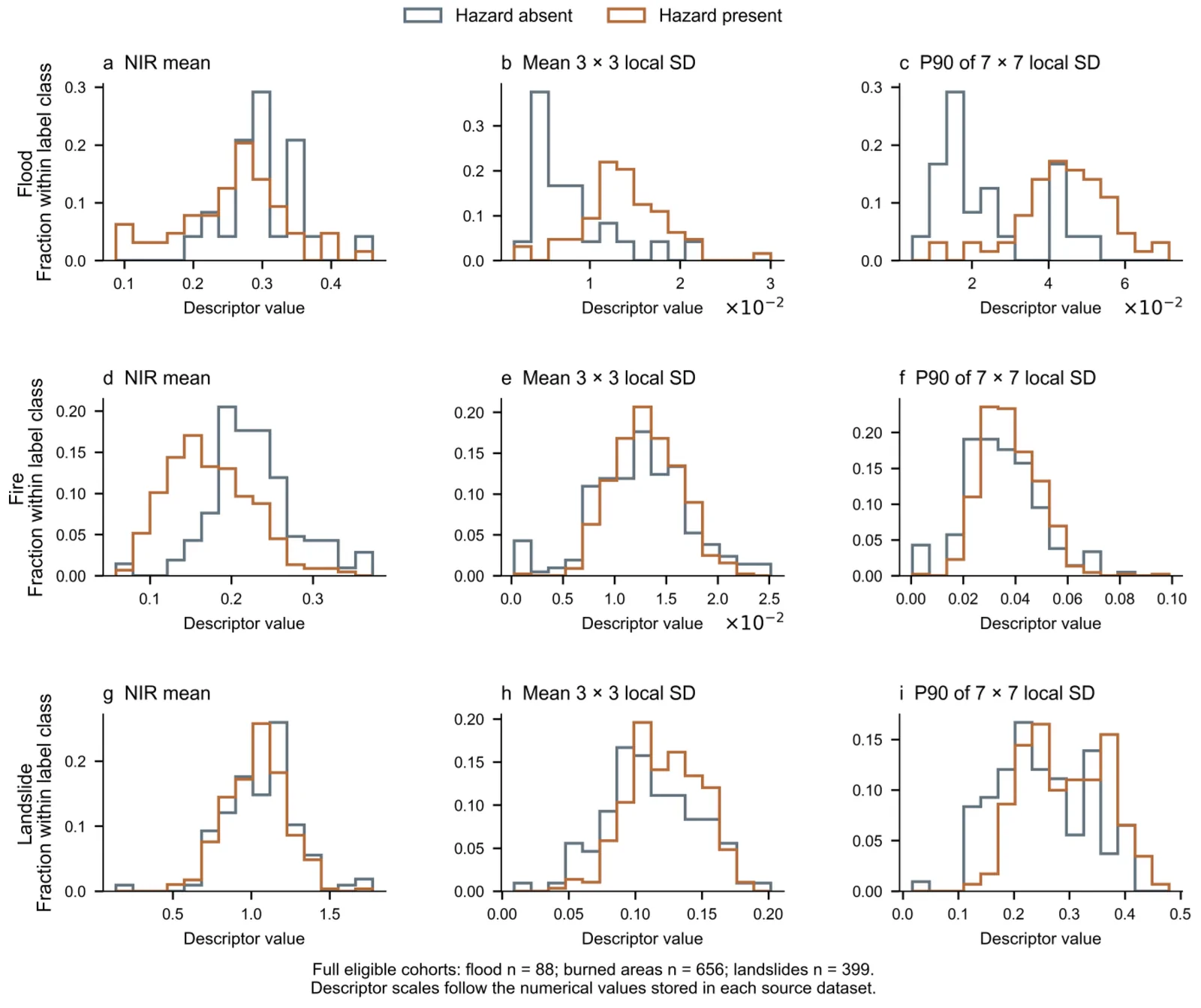
Presence-stratified distributions of three window-level optical descriptors. Columns show B08 mean, mean 3 × 3 local standard deviation, and P90 7 × 7 local standard deviation. Rows show flood (88 patches, 64 present/24 absent), burned area (656 windows, 446/210), and landslide (399 patches, 291/108). Histograms are normalized within each class. All displayed values are finite. Landslide distributions include training, validation and test patches and serve a descriptive purpose. Axes retain each source’s stored numerical scale.

Figure 4 shows the label-stratified distributions of three B08 descriptors from all eligible source samples. The global mean, mean local standard deviation at 3 × 3 pixels, and P90 local standard deviation at 7 × 7 pixels summarize different aspects of each window. Within-class normalization separates distribution shape from class frequency. The distributions illustrate the numerical inputs available to each expert; their source-dependent scales and overlaps motivate evaluation of combined descriptors rather than a common single-feature cutoff.

**Figure 5:**
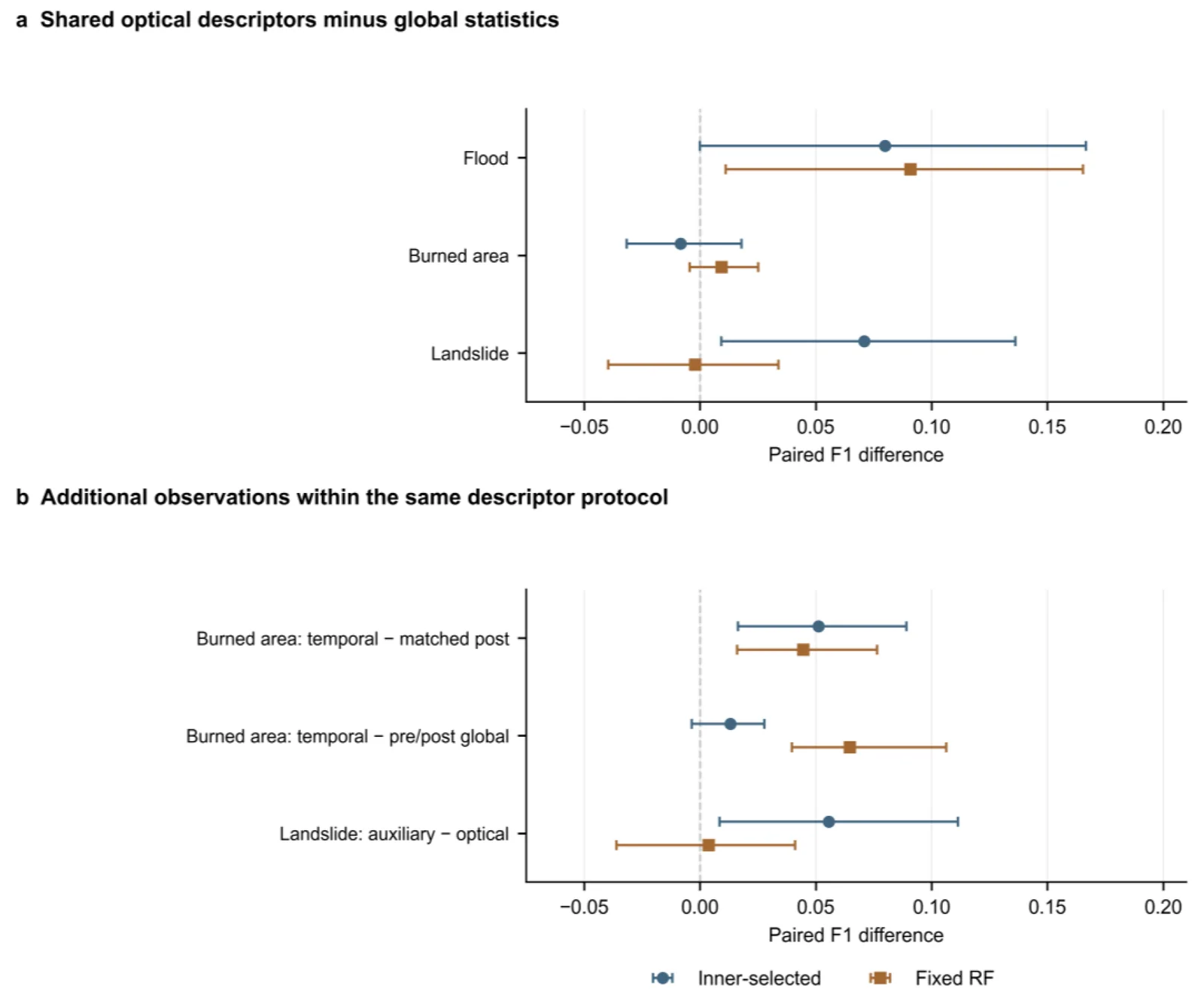
Paired F1 differences under inner-selected and fixed Random Forest evaluation. (**a**) Shared optical descriptors minus global-only descriptors. (**b**) Burned area temporal descriptors minus matched post-only or paired date global descriptors, and landslide optical-plus-auxiliary descriptors minus shared optical descriptors. Positive differences favor the first representation. Intervals are the existing 95% conditional bootstrap intervals from 2000 resamples of fixed predictions. Flood and burned area resampling uses complete source groups; landslide resampling uses test patches under an independence assumption. Intervals exclude refitting and model selection uncertainty.

Local summaries improved flood F1 over global statistics under both evaluation roles. The increase was 0.0799 for the inner-selected procedure and 0.0909 for fixed Random Forest. The latter had a conditional paired interval of [0.0112, 0.1654] (Figure 5). Across all tasks, F1 and ranking metric differences for the global-only and LBP comparisons depended on the source and classifier role.

Index-based flood references supplied a distinct comparison. The fixed mean MNDWI sign rule and MNDWI statistics classifier test index-specific screening, while the optical and optical-plus-index logistic regression comparisons hold classifier family and candidate budget constant.

### 3.3. Temporal and Auxiliary Information Within the Unified Protocol

The burned area temporal representation improved F1 over matched support post-only descriptors in both roles. The inner-selected increase was 0.0512 [0.0164, 0.0891], and the fixed Random Forest increase was 0.0446 [0.0161, 0.0765]. Under inner selection, false positives decreased from 48 to 31 and false negatives from 82 to 56. The paired date global reference also performed well. Relative to that 84-dimensional reference, the temporal representation’s inner-selected F1 difference was 0.0131 [−0.0036, 0.0278], whereas the fixed Random Forest difference was 0.0647. Thus, the temporal gain is clearest against post-only information; the advantage over an alternative two-date representation depends on the classifier role.

The landslide auxiliary extension increased inner-selected F1 by 0.0556 to 0.8725. The selected Random Forest specification changed between these representations. With the prespecified fixed Random Forest, the increase was 0.0038 [−0.0361, 0.0411]. Fixed Random Forest false positives decreased from 9 to 7, while false negatives increased from 13 to 14. The two evaluation roles identify a representation–classifier interaction in the auxiliary channel gain.

### 3.4. Training Label Controls, Class Composition and Sensitivity

True-label BA and AUC exceeded the empirical P97.5 permutation values for each of the five primary task–representation configurations (Table 4). These controls support label-associated discrimination under a fixed selected configuration. F1 alone gave a different impression in the positive-dominated landslide test subset: shared optical true-label F1 was 0.8169, compared with permutation mean F1 0.8137 and an all-positive reference of 0.8571. Its BA of 0.7067 and AUC of 0.8304 provide complementary evidence of two-class discrimination.

The fixed Random Forest neighborhood-scale comparisons changed the shared optical F1 by approximately −0.0102 to +0.0078 across the three tasks. Area-based target requirements changed both the prediction problem and the retained population. For example, a 5% positive area requirement retained 48 of 88 flood patches, and its F1 of 0.9565 should be compared with 0.7925 for the same retained cohort. It does not describe performance on all 88 patches. The burned area 1 ha sensitivity retained 652 of 656 windows and produced small same-cohort F1 differences under fixed Random Forest.

### 3.5. Cross-Source Response of Independent Experts

Local models with additional official training-pixel supervision reduced activation into flood and burned area experts in all four measured incoming directions (Supplementary Materials). Fire source activation of flood receivers was 24.17% for local evidence and 22.02% with indices; the corresponding encoding-adapted landslide source rates were 71.82% and 34.73%. The local burned area receiver activated on 2.22% of flood source and 28.96% of landslide source inputs. The local models also had lower pooled FP and FN counts in the existing held-out evaluations. Table 5 reports the six-direction COMMON78 references. The landslide comparisons showed a trade-off between lower activation and retention of true positives.

Source-specific numerical conditions materially affected transferred responses, including apparent near-zero activation under direct stored value transfer. The encoding method and selected source presence and source group summaries are provided in Supplementary Materials, Parts C and D, alongside the held-out error comparisons.

## 4. Discussion

### 4.1. What the Unified Framework Establishes

The framework gives three independent recognition tasks the same measurement description and a comparable learning procedure. Global means, dispersions and quantiles describe spectral value distributions. Neighborhood standard deviations add local heterogeneity, while Sobel-magnitude summaries characterize transition strength. Their aggregation supplies spatial information beyond global distributions through explicitly defined operations. The local ablation evaluates the neighborhood and gradient summaries together. This shared representation makes complementary information testable across independently fitted experts. Training-side selection and the fixed Random Forest comparison distinguish the performance of the learning procedure from representation effects under a constant classifier setting.

Water absorption, vegetation disturbance and material exposure motivate the common wavelength selection. The experiments evaluate these channels in the source-specific numerical domains supplied by each dataset. A shared presence rule aligns the logical classification target, while the dataset description specifies the physical size and content of each image unit. Together, these datasets provide complementary tests of the same descriptor and learning protocol under distinct observation conditions.

### 4.2. Why Additional Observations Have Conditional Benefits

Post-event descriptors characterize the observed surface, while descriptors of the post-minus-pre array add a temporal reference for that state. The burned area experiment supports this complementarity through reduced false positives and false negatives relative to matched support post-only inputs. Both model roles show a positive F1 change. The difference from pre/post global concatenation is smaller under inner selection, so the benefit also depends on how temporal information is represented and used by the classifier.

Local optical structure and the auxiliary landslide channels play a different role. Neighborhood variation summarizes spatial heterogeneity within a window, while source auxiliary channels add information that may interact with an already informative optical description. The selected and fixed Random Forest comparisons show how much this interaction matters: a gain under the selection procedure can accompany a small fixed classifier F1 change and a different false positive/false negative trade-off. Reporting both roles preserves that distinction. Physical interpretation of the auxiliary extension requires verified channel units and geospatial provenance, beyond its present numerical evaluation.

### 4.3. Position Relative to Machine Learning and Deep Segmentation

Deep learning studies illustrate how learned spatial representations support fine-grained hazard mapping. Konapala et al. evaluated U-Net with Sentinel-1/2 bands and water indices on 446 hand-labelled Sen1Floods11 images spanning 11 flood events [24]. Gebrehiwot et al. used a VGG-based fully convolutional network to delineate flooded areas in UAV imagery [25]. For burned areas, the FLOGA benchmark compared spectral-index, conventional machine learning and deep learning approaches on bi-temporal imagery [8], while BiAU-Net uses paired Sentinel-2 observations to produce spatial burn masks [9]. Lazzeri et al. examined post-fire land cover change and vegetation recovery using Sentinel-2, PRISMA and UAV observations [26]. Landslide4Sense evaluated 11 segmentation architectures with optical and topographic layers [6]. Together, these studies illustrate complementary choices of observation platform, temporal information and spatial representation for hazard mapping.

The present framework addresses a related output: hazard presence within an image window. It represents each window with explicit spectral and spatial descriptors, then fits an independent classifier for each hazard. This design exposes the contribution of shared and task-specific measurements through controlled ablations. Segmentation networks learn spatial filters end to end and return dense masks, whereas the present experts provide window-level presence decisions. These approaches serve complementary mapping needs, from delineating affected pixels to interpretable recognition of hazard presence under grouped evaluation.

The candidate grid spans linear decision functions and tree ensembles in the same tabular descriptor space. Logistic regression and linear SVM represent linear boundaries, while Random Forest and ExtraTrees capture nonlinear feature interactions. The FLOGA benchmark also evaluates XGBoost and multilayer perceptron models [8], and Prithvi transfers pretrained geospatial representations to task-specific decoders [10]. These families offer complementary model designs for different input and output structures. The present framework isolates the behavior of a shared explicit descriptor with independently fitted hazard experts.

### 4.4. Reliability, Study Scope and Future Tests

Balanced accuracy and negative-class errors complement F1 by showing how the experts distinguish the two classes. Training label permutations assess dependence on true labels for the tested primary configurations. The local comparisons reduce cross-source activation for flood and burned area receivers; the landslide comparisons identify an activation–recall trade-off (Supplementary Materials).

Geographic transfer and negative-class coverage are priorities for further validation. Current estimates concern the evaluated cohorts, including the 100-patch landslide test subset with unverified geographic independence, while burned area groups separate source/overlap units rather than verified events. Future studies should extend the protocol to geographically identified cohorts with more negative examples and verified measurement metadata and compare learned representations under the same presence targets and grouped partitions.

## 5. Conclusions

We developed a unified multispectral feature framework that connects a common optical descriptor with independently trained flood, burned area and landslide experts. Shared global and local operations, explicit support rules and training-side evaluation make representation comparisons reproducible across the three tasks. The controlled results show the value of paired date information for burned area recognition and identify classifier-dependent effects of local and auxiliary information. Negative-class metrics, conditional training label controls and cross-source responses provide a clear account of discrimination and extrapolation. By making the shared and task-specific components explicit, the framework offers a practical basis for evaluating multispectral image window recognition across different hazard datasets.

## Figures and Tables

**Table 1:** Dataset composition, image units and evaluation designs.

Task and Dataset	Image Unit	Samples (Present/Absent)	Grouping and Evaluation
Flood, Sen1Floods11 HandLabeled	512 × 512 pixels	88 (64/24)	Leave one of 11 events out
Burned area, S2-WCD-derived	128 × 128 pixels, 20 m grid	656 (446/210)	Leave one of 23 source/overlap groups out; 41 scenes
Landslide, Landslide4Sense subset	128 × 128 pixels	399 (291/108)	239 train, 60 validation, 100 official-test-partition patches (75/25); final refit on 299

**Table 2:** Shared representations, task-specific extensions and their comparison roles.

Representation	Channels or Operation	Features	Comparison Purpose
Global statistics	Six optical bands, seven summaries per band	42	Global-only reference
Shared optical	Global + local SD + Sobel, six bands	78	Common descriptor protocol
Global + LBP	Global summaries + ten circular LBP bins per band	102	Alternative spatial description
Flood index extension	Shared optical + five index descriptors	143	Flood-specific index information
MNDWI statistics	Seven global MNDWI summaries	7	Index-based classifier reference
Matched post	Shared descriptor on paired image support	78	Same-support temporal reference
Pre + post global	Global descriptors of both dates on paired support	84	Alternative paired date reference
Post + change	Shared post descriptor + descriptor of post-minus-pre values	156	Burned area temporal extension
Optical + auxiliary	Shared optical + two source auxiliary channels	104	Landslide numerical extension

**Table 3:** Held-out performance for the common optical representation and the two task-specific extensions. F1 and BA use held-out class decisions; flood and burned area AUC/AP are macro-averages within defined held-out groups, while landslide values concern one final model. FP/negative and FN/positive give the error counts and their respective class denominators.

Task	Representation	Role	F1	BA	AUC	AP	FP/Negative	FN/Positive
Flood	Shared optical (78)	Inner-selected	0.8504	0.7344	0.7583	0.8420	9/24	10/64
Flood	Shared optical (78)	Fixed RF	0.9091	0.8021	0.7479	0.8424	8/24	4/64
Burned area	Shared optical (78)	Inner-selected	0.8485	0.7938	0.9110	0.9467	48/210	82/446
Burned area	Shared optical (78)	Fixed RF	0.8457	0.7509	0.8852	0.9370	74/210	65/446
Landslide	Shared optical (78)	Inner-selected	0.8169	0.7067	0.8304	0.9440	9/25	17/75
Landslide	Shared optical (78)	Fixed RF	0.8493	0.7333	0.8528	0.9514	9/25	13/75
Burned area	Post + change (156)	Inner-selected	0.8997	0.8634	0.9591	0.9800	31/210	56/446
Burned area	Post + change (156)	Fixed RF	0.8904	0.8123	0.9272	0.9672	60/210	40/446
Landslide	Optical + auxiliary (104)	Inner-selected	0.8725	0.7533	0.8627	0.9542	9/25	10/75
Landslide	Optical + auxiliary (104)	Fixed RF	0.8531	0.7667	0.8704	0.9580	7/25	14/75

**Table 4:** Training label permutation controls for the five primary configurations under the inner-selected role. The selected configuration remains fixed; training labels, preprocessing, weights and estimator fitting are recomputed over 20 repetitions. P2.5–P97.5 is an empirical permutation range. BA is based on held-out class decisions; AUC aggregation follows Table 3.

Task	Representation	Metric	True Labels	Permutation Mean	Permutation P2.5 to P97.5
Flood	Shared optical (78)	BA	0.7344	0.4663	0.3059 to 0.5897
Flood	Shared optical (78)	AUC	0.7583	0.5518	0.3175 to 0.7206
Burned area	Shared optical (78)	BA	0.7938	0.4985	0.4410 to 0.5550
Burned area	Shared optical (78)	AUC	0.9110	0.4911	0.4189 to 0.5855
Landslide	Shared optical (78)	BA	0.7067	0.5040	0.4127 to 0.5733
Landslide	Shared optical (78)	AUC	0.8304	0.4730	0.2601 to 0.6756
Burned area	Post + change (156)	BA	0.8634	0.4995	0.4275 to 0.5454
Burned area	Post + change (156)	AUC	0.9591	0.5038	0.4055 to 0.6212
Landslide	Optical + auxiliary (104)	BA	0.7533	0.5080	0.4323 to 0.5670
Landslide	Optical + auxiliary (104)	AUC	0.8627	0.4602	0.2696 to 0.6405

**Table 5:** Six-direction COMMON78 responses after conversion of the documented optical encoding into each receiving model’s training coordinates. Each value is the mean positive response rate across the receiving model references for the stated unique source cohort. Both model roles are retained; receiving model references are repeated responses rather than independent source samples. Receiving hazard ground truth is unavailable.

Source	Receiving Expert	Unique Source Samples	Receiving Model References per Role	Inner-Selected Response	Fixed RF Response
Flood	Burned area	88	23	0.2554	0.3414
Flood	Landslide	88	1	0.8636	0.8409
Burned area	Flood	656	11	0.9234	0.9159
Burned area	Landslide	656	1	0.8598	0.8369
Landslide	Flood	100	11	0.9773	0.9809
Landslide	Burned area	100	23	0.7217	0.8109

## Data Availability

Dataset release information and original resources are available from the Sen1Floods11 repository (https://github.com/cloudtostreet/Sen1Floods11, accessed on 23 October 2026), the S2-WCD IEEE DataPort record (https://doi.org/10.21227/2t8j-t191), and the Landslide4Sense repository (https://github.com/iarai/Landslide4Sense-2022, accessed on 23 October 2026). The present study uses the source subsets and processing described in Section 2. Study-specific sample identifiers, group membership and derived numerical results are retained by the authors and can be supplied to the editor or reviewers on request.

## Supplementary Materials

The following supporting information can be downloaded at: https://www.mdpi.com/article/10.3390/s26216942/s1, The Supplementary Materials provide shared descriptors, index adapters, preprocessing, classifier settings (Table S1), selection and interval definitions (Part A), local evidence methods (Part B), numerical encoding conversion and evaluation (Part C), and the associated outcome summaries (Part D).

## Abbreviations

The following abbreviations are used in this manuscript:

AP	Average precision
AUC	Area under the receiver operating characteristic curve
BA	Balanced accuracy
BSI	Bare soil index
F1	F1 score
FN	False negative
FP	False positive
FPR	False positive rate
JRC	Joint Research Centre
LBP	Local binary pattern
MNDWI	Modified normalized difference water index
NBR	Normalized burn ratio
NDMI	Normalized difference moisture index
NDVI	Normalized difference vegetation index
NIR	Near-infrared
P10/P25/P50/P75/P90	10th/25th/50th/75th/90th percentiles
RF	Random Forest
S2-WCD	Sentinel-2 Wildfire Change Detection
SD	Standard deviation
SVM	Support vector machine
SWIR	Shortwave infrared
TN	True negative
TP	True positive
