# Supplementary material for: A Unified Multispectral Feature Framework for Independent Recognition of Floods, Burned Areas, and Landslides
Source: Sensors (Basel). 2026 Nov 3;26(21):6942. doi: 10.3390/s26216942 (PMC13766413; doi:10.3390/s26216942)
Supplement: Supplementary file 1 [file sensors-26-06942-s001.zip › sensors-4605897-supplementary.pdf]

sensors

MDPI

Supplementary Materials

Part A Shared representations and evaluation

Study cohorts and presence targets

The three source-specific presence tasks comprise 88 flood windows (64 positive/24 negative), 656 burned-area windows (446/210), and 399 Landslide4Sense patches (291/108). Presence requires at least one valid annotated target pixel; label support is distinct from image support. The 41 burned-area scenes form 23 conservative source/observed-overlap groups, not verified events. Landslide model selection uses 239 training and 60 validation patches, followed by refitting on their 299-member union. Testing uses a prespecified 100-patch subset of the official test partition.

Source-specific inputs and shared optical descriptors

The shared optical order is B02, B03, B04, B08, B11 and B12. Flood digital numbers are divided by 10,000; stored burned-area and Landslide4Sense values are unchanged. A common physical reflectance scale is not established, and the two auxiliary Landslide4Sense channels have unresolved units and geospatial meaning.

The shared descriptor Ψ(X,M) contains 78 values. Each of the six bands contributes seven global descriptors: mean, population standard deviation (ddof=0), and P10/P25/P50/P75/P90, giving 42 values. The remaining 36 are bandwise means and P90s of local standard deviation in 3 × 3 and 7 × 7 neighborhoods and Sobel magnitude. Local stencils require complete valid support, exclude array boundaries and yield missing values when support is empty. Pixel-based neighborhoods have source-dependent ground footprints.

Hazard-specific extensions and model fitting

The burned-area extension contains 156 values, [Ψ(Xpost,Mpair),Ψ(Xpost-Xpre,Mpair)], using identical paired support for both blocks. The second block describes pixelwise temporal differences. Comparators use post-only descriptors (78 values) or concatenated pre/post global descriptors (84 values). The landslide extension adds 26 descriptors from the two stored auxiliary channels, giving 104 values.

Table S1 defines the 14-candidate grid. Training-only median imputation retains entirely missing columns; linear models also use training-weighted standardization. Base sample weights are 1/n_g within group, or 1 without groups, normalized within class and divided by their overall mean. Estimators receive these weights with class_weight=None.

Flood and burned-area candidates are ranked by pooled inner out-of-fold group-equal balanced accuracy, then mean group-equal average precision across inner folds, and finally lexicographic identifier. Landslide candidates use the same ranking priorities on the 239/60 training/validation split, followed by refitting on 299 members. Held-out evaluation data do not select models. The fixed comparator is a 300-tree Random Forest with minimum leaf size 3 and square-root feature sampling. LinearSVC uses its native decision boundary of 0; other estimators use a positive-class score of 0.5. Scores are uncalibrated.

Secondary operating points use inner out-of-fold scores for flood and burned area and the 60-patch validation scores for landslide. Candidates are 101 equally spaced score quantiles plus the primary boundary, ranked by balanced accuracy with inverse-group-size weights where groups exist. LinearSVC scores are divided by each model's training-

margin interquartile range without centering; the final model uses its own training scale. Ties within 10^{-12} favor the boundary closest to the primary threshold, then the higher threshold. This scaling does not calibrate probabilities.

Evaluation and diagnostic summaries

Evaluation leaves out 11 flood event groups or 23 burned-area source/overlap groups; landslide uses its 100-patch test subset. Confusion summaries use held-out decisions. AUC/AP are averaged over groups containing both classes (7/11 flood and 23/23 burned-area groups), without pooling margins across folds. Single-class group metrics remain undefined.

Training-label controls use 20 permutations (seeds 42000–42019) per evaluation unit. Only training labels are permuted; selected configurations remain fixed while preprocessing, weights and estimators are refitted. The 2.5th–97.5th percentiles describe an empirical permutation range, not a confidence interval or full model-selection test. Paired intervals use 2,000 whole-group resamples of fixed held-out predictions without refitting.

Part C describes the numerical-encoding sensitivity analysis and receiving-coordinate conversion for COMMON78 transfer.

Flood index adapter definitions

The flood index adapter uses NDVI = (NIR − Red)/(NIR + Red), MNDWI = (Green − SWIR1)/(Green + SWIR1), NBR = (NIR − SWIR2)/(NIR + SWIR2), NDMI = (NIR − SWIR1)/(NIR + SWIR1), and BSI = [(SWIR1 + Red) − (NIR + Blue)]/[(SWIR1 + Red) + (NIR + Blue)]. Each ratio requires valid six-band support, finite numerator, denominator and output, and a nonzero denominator. Ratio support is computed independently of the six-band descriptors. Global and local ratio summaries add 65 values, giving 143, without index-fraction thresholds. The separate MNDWI adapter uses seven global summaries. Its fixed-sign reference predicts presence when mean supported MNDWI is strictly positive; its fitted logistic-regression reference selects $C \in \{0.1,1,10\}$ within training data.

Table S1. Candidate classifier configurations.

**Classifier**	**Candidate settings**	**Number**
Random Forest	300 trees; minimum leaf size {1,3}; max_features {sqrt,0.5}	4
ExtraTrees	300 trees; minimum leaf size {1,3}; max_features {sqrt,0.5}	4
LinearSVC	C {0.1,1,10}; initial max_iter 10,000	3
Logistic regression	C {0.1,1,10}; max_iter 5,000	3

For tree models, max_features=sqrt selects a square-root-sized subset; 0.5 selects half the features, subject to integer rounding. The grid contains 4 + 4 + 3 + 3 = 14 candidates.

Both tree families use Gini impurity, unrestricted depth, minimum split size 2, seed 42 and one thread. Random Forest uses bootstrap sampling; ExtraTrees does not. Neither uses pruning, out-of-bag estimation, a leaf-count cap or additional class weights.

LinearSVC uses L2 regularization, squared-hinge loss, dual=auto and an intercept. Logistic regression uses liblinear, L2 regularization and an intercept. Both use $C \in \{0.1,1,10\}$, tolerance 0.0001 and seed 42, with initial iteration limits of 10,000 and 5,000,

respectively. A prespecified LinearSVC retry raises the limit to 100,000 without changing other settings; two retries occurred. Held-out performance did not remove candidates.

Part B Local evidence models

Local cascades test additional official pixel-mask supervision while retaining the primary patch-supervised experts and all comparison branches. Repeated cohort use makes these comparisons retrospective development evidence rather than independent confirmation on a new source.

Pixel inputs comprise six optical bands and their local standard deviations in 3 × 3 and 7 × 7 neighborhoods (18 features). Index-augmented variants add NDVI, MNDWI, NBR, NDMI and BSI (23 features), using the formulas in Part A. Pixel inputs require finite six-band support and complete neighborhoods; undefined indices remain missing.

The pixel Random Forest uses 64 trees, depth 16, minimum leaf size 8 and square-root feature sampling. Its 15 output summaries are mean, standard deviation, P90/P95/P99/P99.9, maximum, mean of the highest 0.1%, and proportions ≥0.5/0.8/0.95. They also include largest eight-connected component fractions at 0.5/0.8 and maximum fully supported 3 × 3 and 7 × 7 neighborhood means. The patch-stage Random Forest uses 300 trees, minimum leaf size 3 and square-root feature sampling.

For each held-out flood event, three group-separated inner pixel models generate out-of-fold summaries for training the patch classifier. Each evaluation window is processed by all three pixel models and the same patch classifier; their three patch scores are averaged.

The burned-area candidate uses post-event six-band inputs and the same 18-to-15 cascade. Each of its 23 outer source groups uses three disjoint inner folds for patch-stage training and averages the three evaluation scores. It does not use the 156-dimensional temporal descriptor.

Both stages use seed 42, one thread, class_weight=None and their own training-fitted median imputer, with zero fill for entirely missing columns. Official training masks supply pixel labels under the corresponding hazard definition. Sampling retains at most 128 eligible pixels per patch and class, without replacement, using an ID/class/seed-42-derived stream. Pixel weights equalize group-class mass, then patch mass within each combination and pixel mass within each patch-class. Patch weights follow Part A; landslide has no verified geographic groups. Patch decisions use scores ≥0.5.

The landslide forward cascade trains its pixel stage on 239 patches and its patch stage on the distinct 60 validation-subset patches. These 60 patches therefore provide second-stage training, not independent validation. The reverse cascade exchanges the 239/60 stage assignments. The reciprocal candidate equally averages both complete-path scores at threshold 0.5, without test-driven branch or weight selection. The 100 test patches enter neither stage, and these cascades do not refit both stages on 299 members.

The landslide index cascade uses 23 pixel inputs and 15 patch summaries. Both index arms use 49,964 sampled training pixels (19,372 positive/30,592 negative). Hard-background sampling preserves all sampled positives and 64 original random negatives per patch, adding 64 official negatives with the highest inherited pixel-model responses. Mining excludes original sampled indices, uses seeded ties and recorded fallback filling, and is confined to the 239 training patches. Test patches and transferred sources are excluded. The context candidate adds COMMON78 to the 15 summaries (93 inputs); its matched reference uses COMMON78 alone. Both patch stages use the same 60 members.

The RBF comparator is kernel ridge regression on 0/1 patch targets, with $K(z,z') = \exp(-\gamma \| z-z' \|^2)$ and no intercept. Its nine candidates combine $\alpha \in \{0.1,1,10\}$ and $\gamma \in \{0.1/78,1/78,10/78\}$. Training-only median imputation, weighted standardization and

sample weights follow Part A. Flood and burned-area selection uses three grouped inner folds; landslide uses 239/60 selection and 299-member refitting before its 100-patch test. Ranking follows balanced accuracy, average precision and identifier. Presence requires a score ≥0.5; scores are uncalibrated and need not lie in [0,1]. Transfers use the encoding convention in Part C.

Part C Numerical encoding and evaluation

A Landslide4Sense repository contributor documents division by a fixed 14-channel mean vector (https://github.com/iarai/Landslide4Sense-2022/issues/4). Multiplying the 12 optical channels of the 299 training/validation arrays by these means recovered an integer lattice with maximum residual 9.10×10^{-13}. This confirms numerical encoding, not atmospheric-processing equivalence or auxiliary-channel physical units.

For the six shared bands, the coefficients converting stored Landslide4Sense values to nominal DN/10,000 are [824.63171476, 663.41636217, 445.17289745, 1941.32229668, 1113.98338755, 519.90397929]/10,000. Transfer multiplies each source channel by its coefficient and divides by the receiving-model coefficient. The same positive factor scales its seven global and six local COMMON78 summaries. Direct calculations on nonempty synthetic and real training arrays verified this algebra; all 70 original model references retained identical in-domain predictions.

Flood inputs retain DN/10,000; burned-area inputs retain their stored derived values because their processing lineage is incomplete. Comparisons remain conditional on these numerical conventions. The metadata-based adapter converts coordinates without classifying sources, routing experts or removing inputs.

The fire-to-flood comparison uses 656 windows in 23 source groups: 210 without and 446 with burn-label presence. Each window is evaluated by 11 fixed flood receiver folds, giving 7,216 predictions per arm. These are repeated predictions on 656 windows, not independent observations. Activation is summarized by source-presence strata.

Activation summaries comprise window-weighted means across fixed receiver models and equally weighted means across the 23 source groups. Comparisons pair identical windows and receiver folds. Conditional intervals use 2,000 paired source-group bootstrap resamples (seed 42), with models fixed. They exclude uncertainty from refitting, method development and new-source sampling; repeated predictions do not establish independent-model replication or confirmatory significance.

Part D Outcome summary

Flood-receiver activation on burned-area inputs was 91.59% for fixed RF, 24.17% for local evidence and 22.02% with indices. Corresponding landslide-source rates after encoding conversion were 98.09%, 71.82% and 34.73%. On 88 held-out flood patches, FP/FN counts were 8/4, 7/3 and 6/2, respectively.

The local burned-area receiver activated on 2.22% of flood-source and 28.96% of landslide-source inputs, versus COMMON78 inner-selected references of 25.54% and 72.17%. On 656 held-out windows, local F1/recall were 0.9626/0.9529 with FP/FN 12/21; temporal-selected values were 0.8997/0.8744 with FP/FN 31/56. Local group-macro F1 was 0.9457, with FG18 lowest at 0.5000; FG22 had five false positives among five negatives. Despite lower pooled FPR, local group-macro FPR was 0.0879 versus 0.0855 for the temporal comparator.

For local flood receivers, worst all-presence source-group activation remained 66.48% without indices and 62.50% with indices. Somalia was the highest-activation receiver fold (46.95%/33.84%). In the no-burn stratum, the single FG12 window activated under all 11 receiver folds in both variants.

Landslide local evidence yielded F1 0.8667 and FP/FN 10/10 with high incoming activation. Context increased F1 to 0.9125 and fire-source activation to 89.18%. Hard-background and reciprocal candidates did not meet their fixed combined utility criteria. RBF reduced flood/fire activation to 2.27%/9.15%, but missed 28 of 75 positive test patches (F1 0.7581), precluding adoption as an across-source improvement.

Transferred inputs lack receiving-hazard ground truth, so activation is interpreted alongside in-domain errors and positive retention, not as a receiving-hazard false-positive rate.
